# Three-Dimensional Superresolution Imaging of the FtsZ Ring during Cell Division of the Cyanobacterium *Prochlorococcus*

**DOI:** 10.1128/mBio.00657-17

**Published:** 2017-11-21

**Authors:** Riyue Liu, Yaxin Liu, Shichang Liu, Ying Wang, Kim Li, Ning Li, Daiying Xu, Qinglu Zeng

**Affiliations:** a Division of Life Science, The Hong Kong University of Science and Technology, Hong Kong, China; b Environmental Science Programs, The Hong Kong University of Science and Technology, Hong Kong, China; c Nanobioimaging Ltd., Hong Kong, China; d HKUST Shenzhen Research Institute, Shenzhen, China; Oregon State University

**Keywords:** FtsZ ring, *Prochlorococcus*, cell division, cyanobacteria, superresolution imaging

## Abstract

Superresolution imaging has revealed subcellular structures and protein interactions in many organisms. However, superresolution microscopy with lateral resolution better than 100 nm has not been achieved in photosynthetic cells due to the interference of a high-autofluorescence background. Here, we developed a photobleaching method to effectively reduce the autofluorescence of cyanobacterial and plant cells. We achieved lateral resolution of ~10 nm with stochastic optical reconstruction microscopy (STORM) in the sphere-shaped cyanobacterium *Prochlorococcus* and the flowering plant *Arabidopsis thaliana*. During the cell cycle of *Prochlorococcus*, we characterized the three-dimensional (3D) organization of the cell division protein FtsZ, which forms a ring structure at the division site and is important for cytokinesis of bacteria and chloroplasts. Although the FtsZ ring assembly process in rod-shaped bacteria has been studied extensively, it has rarely been studied in sphere-shaped bacteria. Similarly to rod-shaped bacteria, our results with *Prochlorococcus* also showed the assembly of FtsZ clusters into incomplete rings and then complete rings during cell division. Differently from rod-shaped bacteria, the FtsZ ring diameter was not found to decrease during *Prochlorococcus* cell division. We also discovered a novel double-Z-ring structure, which may be the Z rings of two daughter cells in a predivisional mother cell. Our results showed a quantitative picture of the *in vivo* Z ring organization of sphere-shaped bacteria.

## IMPORTANCE

Superresolution microscopy has not been widely used to study photosynthetic cells due to their high-autofluorescence background. Here, we developed a photobleaching method to reduce the autofluorescence of cyanobacteria and plant cells. After photobleaching, we performed superresolution imaging in the cyanobacterium *Prochlorococcus* and the flowering plant *Arabidopsis thaliana* with ~10-nm resolution, which is the highest resolution in a photosynthetic cell. With this method, we characterized the 3D organization of the cell division protein FtsZ in *Prochlorococcus*. We found that the morphological variation of the FtsZ ring during cell division of the sphere-shaped cyanobacterium *Prochlorococcus* is similar but not identical to that of rod-shaped bacteria. Our method might also be applicable to other photosynthetic organisms.

## INTRODUCTION

Superresolution imaging methods have enabled researchers to visualize subcellular structures and protein interactions in many organisms; however, they have not been widely used in photosynthetic cells, such as cyanobacteria, algae, and plant cells with chloroplasts (1–3). Major superresolution microscopy methods include structured illumination microscopy (SIM), stimulated emission depletion microscopy (STED), stochastic optical reconstruction microscopy (STORM), and photoactivated localization microscopy (PALM) (reviewed in reference 4). Although SIM has been used to study photosynthetic cells (1–3), its lateral resolution is only ~100 nm and is much lower than that of STED, STORM, and PALM, which can be as good as 10 nm (5). The axial resolution of SIM (~250 nm) is also lower than those of STED (150 to 600 nm), STORM (~50 nm), and PALM (~50 nm) (5, 6). The high resolution of STED, STORM, and PALM demands much higher laser power than SIM (4, 5), which causes a strong fluorescence background in cells with autofluorescence (1). Therefore, STED, STORM, and PALM have not been applied in photosynthetic cells, although they have been used to study plant cells without chloroplasts (1, 3).

The autofluorescence of oxygenic photosynthetic organisms originates mainly from pigments associated with photosynthetic complexes, and chlorophyll fluorescence from photosystem II predominates (7). During prolonged exposure to high light, photosynthetic organisms have evolved photochemical and nonphotochemical mechanisms to bring the excited pigment molecules to their ground state (8). During these processes, the fluorescence yield of pigments is decreased, which is termed fluorescence quenching (8). In fluorescence microscopy, photobleaching has been commonly used to quench fluorescent fusion proteins or dyes to visualize multiple biomarkers sequentially (9), and this approach can also quench autofluorescence to improve the signal-to-noise ratio. Thus, photobleaching prior to immunostaining is considered to be a highly desirable solution to visualize photosynthetic cells using superresolution microscopy. In this work, photobleaching enabled us to use STORM to study the *in vivo* organization of the cell division protein FtsZ in the photosynthetic cyanobacterium *Prochlorococcus*.

FtsZ is a highly conserved tubulin-like GTPase that is essential for both bacterial (10) and chloroplast (11, 12) division. FtsZ monomers polymerize into protofilaments (13) to form a ring structure *in vivo* at the division site, which is called the Z ring (10). The function of the Z ring during cytokinesis is still highly debatable. While some evidence suggests that Z ring contraction during cell division provides the constrictive force for cytokinesis (13, 14), recent evidence suggests that the Z ring may mainly act as a cytoskeletal scaffold to recruit other proteins for septal cell wall synthesis (15–17). Two main models have been proposed for the *in vivo* Z ring organization. The patchy band model proposes that FtsZ protofilaments do not have strong lateral interactions and assemble into a discontinuous band at midcell (18). On the other hand, the lateral interaction model proposes that FtsZ protofilaments interact to form a continuous ring or helix (19). The patchy band model is supported by three-dimensional (3D) PALM studies of the rod-shaped bacteria *Caulobacter crescentus* (20) and *Escherichia coli* (15). While rods can assemble the Z ring only at midcell (21), sphere-shaped bacteria (cocci) can assemble the Z ring at an infinite number of theoretical division planes, and their Z rings are also larger than those of rods (21). Therefore, the Z ring organization of cocci might be different from that of rods (21, 22). A 3D SIM study of *Staphylococcus aureus* (23) is so far the only superresolution study conducted on the Z rings of sphere-shaped bacteria. However, it failed to prove the patchy band or the lateral interaction model conclusively due to its low resolution (20).

In the present study, we applied STORM to characterize the *in vivo* Z ring organization of the sphere-shaped unicellular cyanobacterium *Prochlorococcus*. With a cell diameter of 500 to 700 nm (24) and an estimated global population of ~10^27^ cells (25), *Prochlorococcus* is the smallest and most abundant photosynthetic organism on earth (26). We first showed that photobleaching can effectively reduce the autofluorescence of *Prochlorococcus* and also cells of the plant *Arabidopsis thaliana*. Next, using 3D STORM, we imaged the *in vivo* Z ring organization of synchronized *Prochlorococcus* cells at different stages of the cell cycle. In addition, we measured Z ring diameters during cell division.

## RESULTS

### Photobleaching reduces the autofluorescence of the cyanobacterium *Prochlorococcus* and cells of the plant *Arabidopsis thaliana*.

**FIG 1:**
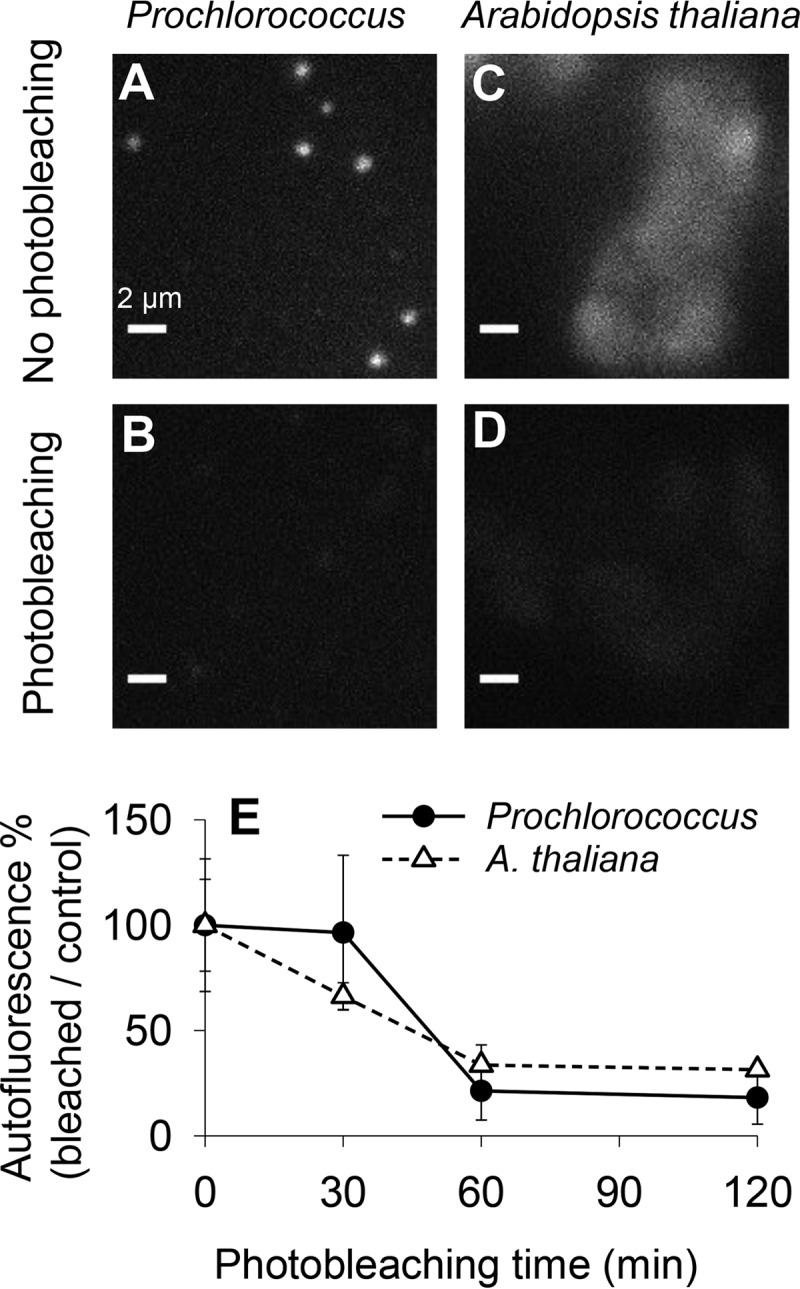
Photobleaching of the cyanobacterium *Prochlorococcus* and the plant *Arabidopsis thaliana*. (A to D) Fixed *Prochlorococcus* MED4 (A and B) and *A. thaliana* (C and D) cells were not photobleached (A and C) or were photobleached by exposure to white light of high intensity for 60 min (B and D). Cells immersed in imaging buffer were excited by a 750-nm laser at the same intensity as that used for STORM imaging, and their autofluorescence was measured. Bars, 2 µm. (E) After photobleaching for 0, 30, 60, and 120 min, autofluorescence of the photobleached cells was measured and normalized to that of the control cells at 0 min. Error bars represent the standard deviations from at least 20 *Prochlorococcus* MED4 cells or five *A. thaliana* cells.

A low-fluorescence background is crucial for STORM imaging, since STORM achieves single-molecule imaging by stochastically activating individual photoswitchable fluorophores within the diffraction-limited region and then calculating the center of each fluorophore molecule (4). We chose to use the fluorophore Alexa Fluor 750 (absorption maximum, 750 nm) to avoid the absorption spectrum of *Prochlorococcus*, which peaks at 447 and 680 nm (27). Although *Prochlorococcus* has a minimum absorption at wavelengths above 700 nm (27), due to the high intensity of the excitation laser (750 nm) used in STORM, *Prochlorococcus* MED4 cells still showed high autofluorescence within the emission spectrum (777 to 809 nm) of Alexa Fluor 750 (Fig. 1A). We photobleached preserved *Prochlorococcus* MED4 cells by exposing them to white light of high intensity (see Materials and Methods) for 30, 60 (Fig. 1B), and 120 min, respectively. The photobleaching curve showed that the autofluorescence can be reduced by ~80% after 60 min (Fig. 1E). To test whether our photobleaching treatment caused cellular structure damages, we used STORM to visualize the cytoskeleton microtubules of mammalian COS-7 cells, which were imaged in the original STORM studies (6, 28). Our data (see Fig. S1 in the supplemental material) showed that the microtubule structures with and without high-light exposure for 60 min were both similar to those shown in previous studies (6, 28), suggesting that high-light exposure did not affect cytoskeletal structures.

10.1128/mBio.00657-17.1 FIG S1  STORM imaging of microtubules in COS-7 cells. Download FIG S1, PDF file, 0.2 MB. Copyright © 2017 Liu et al. 2017 Liu et al. This content is distributed under the terms of the Creative Commons Attribution 4.0 International license.

Photobleaching can also reduce the autofluorescence of the cells of the plant *Arabidopsis thaliana* (Fig. 1C, D, and E). After photobleaching, we were able to use STORM to visualize the localization of the plant plasma membrane intrinsic protein PIP2a (Fig. S2), which is a water channel (29).

10.1128/mBio.00657-17.2 FIG S2  STORM imaging of plant plasma membrane intrinsic protein 2a (PIP2a; AT3G53420). Download FIG S2, PDF file, 0.1 MB. Copyright © 2017 Liu et al. 2017 Liu et al. This content is distributed under the terms of the Creative Commons Attribution 4.0 International license.

### STORM reveals four Z ring morphologies throughout the cell cycle of *Prochlorococcus.*

**FIG 2:**
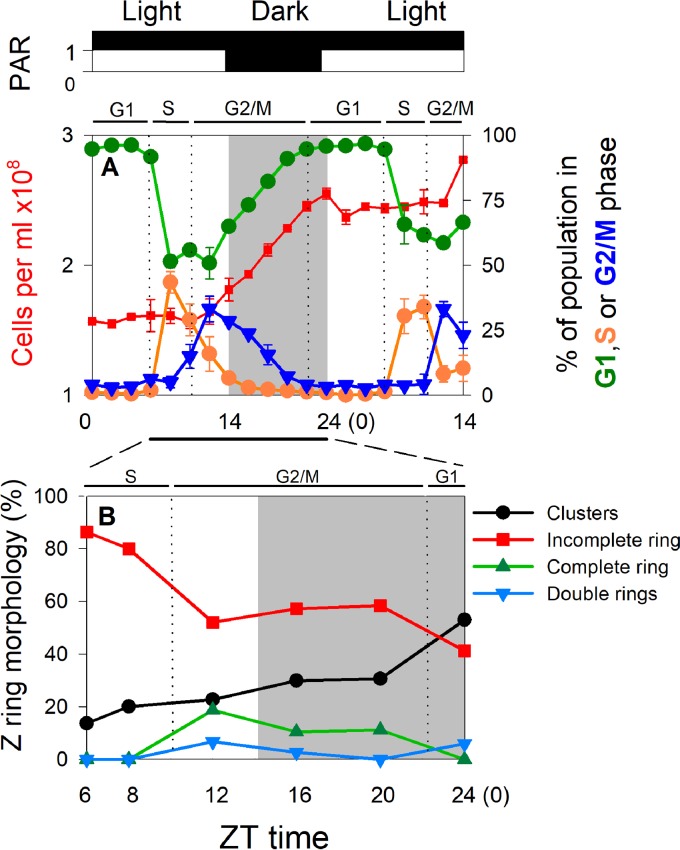
Quantification of Z ring morphologies during the cell cycle of *Prochlorococcus*. (A) *Prochlorococcus* MED4 cells were synchronized to a 14-h light–10-h dark cycle. The period with constant light was from zeitgeber time zero (ZT 0) to 14, and the dark period was from ZT 14 to 24 (0). Cell concentrations and percentages of cells in the G_1_, S, and G_2_/M cell cycle phases were analyzed by flow cytometry. Relative photosynthetically available radiation (PAR) is shown above panel A. Error bars represent the standard deviations from two biological replicates. (B) At ZT 6, 8, 12, 16, 20, and 24, the *in vivo* FtsZ organization of 257 cells was imaged by STORM and the percentages of different Z ring morphologies during the cell cycle of *Prochlorococcus* were manually analyzed.

In order to capture the Z ring morphological changes during the cell cycle of *Prochlorococcus*, we performed 3D STORM imaging of *Prochlorococcus* cells in different cell cycle phases. We synchronized *Prochlorococcus* MED4 cells by growing them under a 14-h light–10-h dark cycle (Fig. 2A), where zeitgeber time zero (ZT 0) corresponds to lights on and ZT 14 corresponds to lights off. Similarly to previous studies (30, 31), *Prochlorococcus* MED4 cells synthesized their DNA from around ZT 6 to 10 (S phase) and divided from around ZT 10 to 22 (G_2_/M phase) (Fig. 2A). Between the G_2_/M phase and the S phase is the G_1_ phase (ZT 22 to 6) (Fig. 2A). Under our growth conditions, *Prochlorococcus* MED4 cells grew at a lower division rate (0.60 ± 0.07 division per day) than previous studies (about one division per day) (30, 31), suggesting that not all the cells in our cultures divided during our experiment. This might be due to different irradiation patterns in the light period (constant low light in our study versus bell-shaped high light in previous studies) (32). In cyanobacteria, the circadian clock allows cell division only at a certain period of a light-dark cycle (33). The circadian gating of cell division is achieved through elevated ATPase activity of the circadian clock protein KaiC, which inhibits Z ring formation at the division site without affecting the FtsZ concentration in a cell (34). Therefore, the nondividing cells in our cultures should contain only FtsZ protofilaments (clusters) and should not affect our observation of the Z ring morphological changes of the dividing cells.

**FIG 3:**
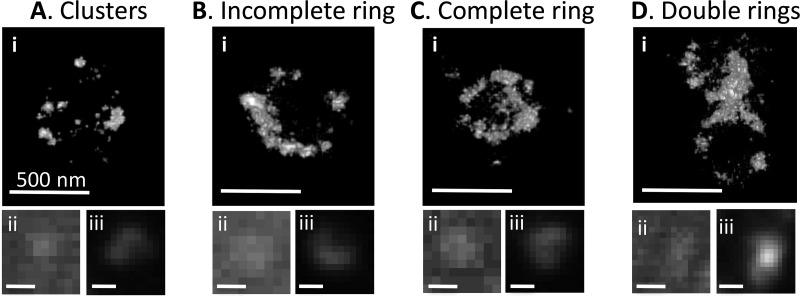
Representative STORM images of four Z ring morphologies. Four Z ring morphologies were found during the cell cycle of *Prochlorococcus*: clusters (A), incomplete ring (B), complete ring (C), and double rings (D). Images of STORM (i), bright-field microscopy (ii), and wide-field fluorescence microscopy (iii) are shown for each cell. Bars, 500 nm. Corresponding 3D images of panels A, B, C, and D are shown in Movies S1, S2, S3, and S4, respectively.

At different cell cycle stages, *Prochlorococcus* MED4 cells were preserved, photobleached to reduce autofluorescence, and immunostained with an anti-FtsZ antibody. This immunostaining method has been routinely used in superresolution imaging studies and reveals a similar Z ring morphology as does the fluorescent FtsZ fusion protein (15, 23). The *in vivo* FtsZ organization of 257 cells was imaged by 3D STORM with spatial resolutions of 9.6 nm in the *x-y* plane and 41.6 nm in the *z* axis (see Materials and Methods). Our sample size is comparable to that of a recent 3D PALM study of 275 synchronized *C. crescentus* cells, but our resolutions are much higher than those of the PALM study (35 nm in *x-y* and 120 nm in *z*) (20). Overall, we classified four distinct Z ring morphologies by their 3D STORM images: clusters, incomplete rings, complete rings, and double rings, all of which were observed as spots or bars under a conventional fluorescence microscope (Fig. 3). Of the 257 imaged cells, 26.8% did not show any obvious ring structure and yet they contained clusters of FtsZ proteins or protofilaments (Fig. 3A; Movie S1). Sixty percent of cells contained an incomplete ring (Fig. 3B; Movie S2), and 10.1% contained a complete ring (Fig. 3C; Movie S3). Similar to the Z rings of *C. crescentus* (20), complete Z rings of *Prochlorococcus* had lots of small gaps in them (Fig. 3C; Movie S3), which is consistent with the patchy band model for *in vivo* Z ring organization (18). These gaps were less likely to result from uneven immunostaining with anti-FtsZ antibodies (15, 23), although we could not exclude the possibility that they were caused by photobleaching-induced damage.

10.1128/mBio.00657-17.4 MOVIE S1  3D image of Fig. 3A. Download MOVIE S1, AVI file, 2.8 MB. Copyright © 2017 Liu et al. 2017 Liu et al. This content is distributed under the terms of the Creative Commons Attribution 4.0 International license.

10.1128/mBio.00657-17.5 MOVIE S2  3D image of Fig. 3B. Download MOVIE S2, AVI file, 3 MB. Copyright © 2017 Liu et al. 2017 Liu et al. This content is distributed under the terms of the Creative Commons Attribution 4.0 International license.

10.1128/mBio.00657-17.6 MOVIE S3  3D image of Fig. 3C. Download MOVIE S3, AVI file, 3 MB. Copyright © 2017 Liu et al. 2017 Liu et al. This content is distributed under the terms of the Creative Commons Attribution 4.0 International license.

### Double Z rings in predivisional mother cells.

**FIG 4:**
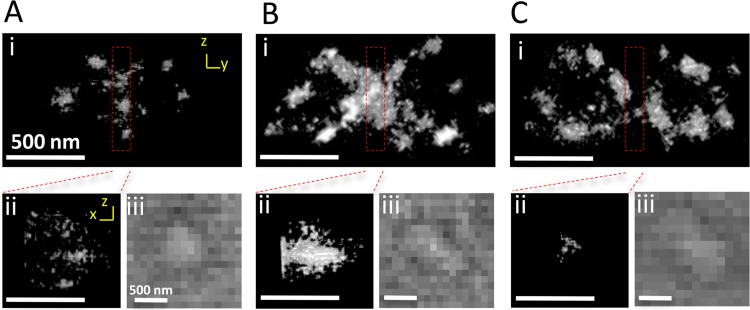
STORM images of double Z rings. Three representative *Prochlorococcus* cells with double Z rings are shown. The mother Z ring was a complete ring (A), a bright spot (B), or absent (C). (i) STORM image in *y-z* plane. (ii) STORM image of cropped midcell mother Z ring in *x-z* plane. (iii) Bright-field image. Bars, 500 nm. Corresponding 3D images of panels A, B, and C are shown in Movies S5, S6, and S7, respectively.

In our STORM images, 3.1% of cells contained double Z rings (Fig. 3D and 4; Movies S4 to S7), and these cells seemed to be elongated (white-field images in Fig. 3D and 4). In the rod-shaped bacterium *E. coli*, Z rings of the two daughter cells sometimes form during the final constriction stage of the mother cell, and they are parallel to the mother Z ring (13, 35). Moreover, replicated chromosomes are segregated and located near the future division sites of daughter cells (36), suggesting that these division sites are active before mother cell division is complete (37). Daughter Z rings were also found in the sphere-shaped cyanobacterium *Synechocystis* sp. strain PCC 6803 and were perpendicular to the mother Z ring, which condensed to a bright spot in the middle of the predivisional mother cell (38). For the first time, our STORM imaging showed the superresolution structures of this double-Z-ring morphology (Fig. 3D and 4). In all the cells that contained double Z rings, daughter Z rings were always perpendicular to the mother Z ring, consistent with the orientation of daughter Z rings in sphere-shaped bacteria (22). We found that the mother Z ring can be a complete ring (Fig. 4A; Movie S5), a bright spot (Fig. 4B; Movie S6) that is similar to *Synechocystis* sp. PCC 6803 (38), or almost absent (Fig. 4C; Movie S7), which might represent the early, middle, and late stages of mother cell division, respectively.

10.1128/mBio.00657-17.7 MOVIE S4  3D image of Fig. 3D. Download MOVIE S4, AVI file, 2.9 MB. Copyright © 2017 Liu et al. 2017 Liu et al. This content is distributed under the terms of the Creative Commons Attribution 4.0 International license.

10.1128/mBio.00657-17.8 MOVIE S5  3D image of Fig. 4A. Download MOVIE S5, AVI file, 2.9 MB. Copyright © 2017 Liu et al. 2017 Liu et al. This content is distributed under the terms of the Creative Commons Attribution 4.0 International license.

10.1128/mBio.00657-17.9 MOVIE S6  3D image of Fig. 4B. Download MOVIE S6, AVI file, 3.3 MB. Copyright © 2017 Liu et al. 2017 Liu et al. This content is distributed under the terms of the Creative Commons Attribution 4.0 International license.

10.1128/mBio.00657-17.10 MOVIE S7  3D image of Fig. 4C. Download MOVIE S7, AVI file, 3.3 MB. Copyright © 2017 Liu et al. 2017 Liu et al. This content is distributed under the terms of the Creative Commons Attribution 4.0 International license.

### Variation of Z ring morphologies during *Prochlorococcus* cell cycle.

Our STORM data set enabled us to quantitatively analyze the variation of different Z ring morphologies during the cell cycle of *Prochlorococcus*. Before cell division in the S phase, incomplete rings were the dominant morphology, followed by clusters, while complete rings and double rings were not observed (Fig. 2B). Entering cell division in the G_2_/M phase, incomplete rings decreased dramatically while clusters increased slightly (Fig. 2B). Meanwhile, complete rings and double rings started to appear (Fig. 2B), which was coincident with the peak of the percentage of cells in the G_2_/M phase (Fig. 2A). From the S phase to the G_2_/M phase (from ZT 8 to 12), the 26% increase of cells containing complete rings or double rings and the 28% decrease of cells containing incomplete rings suggested that incomplete rings were converted to complete rings and double rings during cell division. After cell division in the G_1_ phase, complete rings totally disappeared, and only one cell was observed to contain double rings (Fig. 2B). In contrast, clusters became the dominant Z ring morphology (Fig. 2B), indicating that Z rings were converted back to FtsZ clusters after cell division.

### Diameters of different Z ring morphologies.

**FIG 5:**
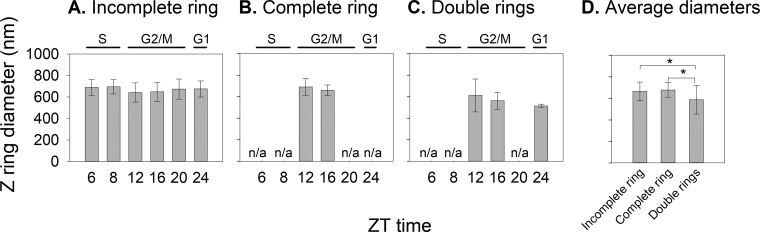
Z ring diameter as a function of the cell cycle. (A to C) Diameters of incomplete rings (A), complete rings (B), and double rings (C) were measured at ZT 6, 8, 12, 16, 20, and 24 during a light-dark cycle, with the S, G_2_/M, and G_1_ cell cycle phases labeled above each graph. If a ring morphology was not seen at a certain time point, its diameter at that time point was not shown (n/a). (D) Average diameters of different Z ring morphologies. Error bars represent the standard deviations from at least two rings. Analysis of variance (ANOVA) was performed for the values shown within each graph. An asterisk indicates a *P* value of <0.05 by ANOVA *post hoc* tests.

We calculated the average diameters of different Z ring morphologies in our data set and also compared the diameters of the same Z ring morphology during the cell cycle (Fig. 5). Cells containing FtsZ clusters were excluded from this analysis since they did not show any obvious ring morphology. We found that the average diameters of incomplete rings (664 ± 86 nm) and complete rings (678 ± 69 nm) were similar (Fig. 5D) (*P* > 0.05 by analysis of variance [ANOVA]), and both were comparable with the 500- to 700-nm diameter of *Prochlorococcus* cells (24). As expected, incomplete rings and complete rings were larger than daughter rings (588- ± 132-nm diameter for one ring) in the cells containing double rings (Fig. 5D) (*P* < 0.05 by ANOVA), probably because one mother cell is bigger than one daughter cell in a predivisional mother cell. During cell division in the G_2_/M phase, the diameter of the same Z ring morphology did not change significantly (Fig. 5A to C) (*P* > 0.05 by ANOVA). Our results are in contrast to studies of the rod-shaped bacteria *E. coli* and *C. crescentus*, which showed that complete rings were smaller than incomplete rings (15, 20). Also different from our results, the Z ring diameter of *E. coli* decreased from ~550 nm to ~250 nm during cell division (15) and that of *C. crescentus* decreased from ~550 nm to ~330 nm (20). We suspected that the different trends of Z ring diameters between sphere-shaped *Prochlorococcus* and rod-shaped bacteria might be due to their different cell shapes (see Discussion for further explanation).

## DISCUSSION

In this study, we developed a photobleaching method to reduce the autofluorescence of the cyanobacterium *Prochlorococcus* and the flowering plant *A. thaliana* (Fig. 1). After photobleaching, 3D STORM imaging of *Prochlorococcus* (Fig. 3 and 4) and *A. thaliana* (see Fig. S2 in the supplemental material) was achieved with spatial resolutions of 9.6 nm in the *x-y* plane and 41.6 nm in the *z* axis. To our knowledge, this is the highest resolution that has been achieved in a photosynthetic cell. Our photobleaching method is not only useful for cyanobacteria and plants but is probably also useful for algae and organisms containing proteorhodopsin and bacteriochlorophyll.

**FIG 6:**
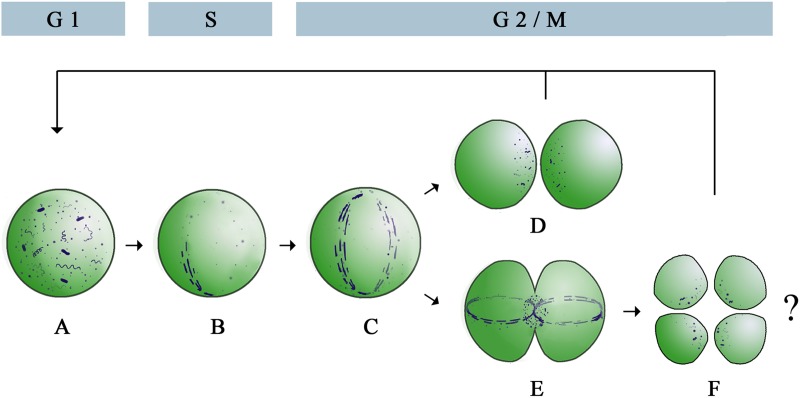
Proposed model of Z ring assembly during the cell cycle of *Prochlorococcus*. During the G_1_ phase, FtsZ protofilaments are distributed in the cell as clusters (A). FtsZ protofilaments form an incomplete ring at midcell in the S phase (B) and extend into a complete ring during cell division in the G_2_/M phase (C). After cell division, the complete ring is disassembled and is converted back into clusters (D). In some mother cells, one Z ring assembles at midcell of each daughter cell and is perpendicular to the mother Z ring (E). After cell division, one mother cell is probably divided into four daughter cells (F), instead of two daughter cells during normal growth (D).

The variation of different Z ring morphologies during the *Prochlorococcus* cell cycle allows us to propose a Z ring assembly model for sphere-shaped bacteria (Fig. 6). In this model, FtsZ protofilaments are distributed as clusters in the G_1_ phase (Fig. 6A) and assemble into an incomplete ring at midcell in the S phase (Fig. 6B). During cell division in the G_2_/M phase, a complete ring forms (Fig. 6C) and is disassembled into FtsZ clusters after one mother cell divides into two daughter cells (Fig. 6D). Occasionally, Z rings form in the two daughter cells of a predivisional mother cell and are perpendicular to the mother Z ring (Fig. 6E). In these rare events, one mother cell might divide into four daughter cells (Fig. 6F).

The Z ring morphological variation during cell division of sphere-shaped *Prochlorococcus* is similar but not identical to that of rod-shaped *C. crescentus* (20) and *E. coli* (15). During cell division of these three bacteria, the processes of assembly of FtsZ clusters into incomplete rings and then complete rings are similar (Fig. 2B) (15, 20). Additionally, the Z rings of these three bacteria all contain many small gaps and are not continuous structures (Fig. 3) (15, 20), supporting the patchy band model for *in vivo* Z ring organization (18). However, we did not observe Z ring contraction during *Prochlorococcus* cell division, but it has been observed in *C. crescentus* (20) and *E. coli* (15). We can think of two possible explanations. One possibility is that the Z ring of *Prochlorococcus* contracts rapidly at the end of cell division, but we did not capture these structures because of infrequent sampling. Although the highly condensed mother Z rings in the cells containing double Z rings might be contracted Z rings (Fig. 4B), they were not observed in the cells containing a single Z ring. The second possibility is that the Z ring of *Prochlorococcus* does not contract during cell division. During cell division, rod-shaped bacteria elongate the lateral cell wall and also synthesize cell wall at midcell (a septum) to divide the mother cell into two daughter cells; however, sphere-shaped bacteria mainly synthesize the septum (22). We hypothesize that a noncontractile Z ring might be used as a scaffold for septum synthesis in sphere-shaped bacteria, while a contractile Z ring might be used for both septum and lateral cell wall synthesis in rod-shaped bacteria. Future studies are needed to explore whether the Z ring functions in rods and cocci are different. Since chloroplasts of algae and plants originated from endosymbiosis of an ancestral cyanobacterium (39) and they all use FtsZ to divide (40, 41), our method may help obtain more insights into the assembly process and function of Z ring in chloroplasts.

## MATERIALS AND METHODS

### *Prochlorococcus* growth and cell cycle analysis.

The axenic *Prochlorococcus* strain MED4 was grown in Port Shelter (Hong Kong) seawater-based Pro99 medium (42). Batch cultures were maintained at 23°C under a 14-h light–10-h dark cycle (35 μmol photons m^−2^ s^−1^ in the light period) for at least 3 months prior to superresolution microscopy. We followed a published protocol to determine the percentage of cells in each cell cycle phase (30). Briefly, preserved *Prochlorococcus* cells were stained with SYBR green (Invitrogen) and analyzed using a flow cytometer (BD FACSCalibur) with the ModfitLT software.

### Fixation and photobleaching of *Prochlorococcus* cells.

In order to preserve *Prochlorococcus* MED4 cells prior to photobleaching, freshly prepared paraformaldehyde and glutaraldehyde were added to 1 ml log-phase culture to final concentrations of 2.5% and 0.05%, respectively. After incubation at room temperature for 20 min, the culture was spun down at 13,500 × *g* for 1 min, and the cell pellet was resuspended in ~100 µl remaining supernatant. Cells were then loaded onto a coverslip (catalog no. 0111580; Marienfeld) precoated with polystyrene beads and poly-l-lysine and kept on ice for 30 min. Cells on the coverslip were briefly rinsed with phosphate-buffered saline (PBS) (10 mM Na_2_HPO_4_, 1.8 mM KH_2_PO_4_, 137 mM NaCl, 2.7 mM KCl, pH 7.4) and permeabilized in 0.05% Triton X-100, 0.2 mg/ml lysozyme, 10 mM EDTA, and 10 mM Tris (pH 8.0) for 20 min at 37°C. After rinsing with PBS, each coverslip was covered by 50 µl blocking buffer (0.2% Triton X-100 and 3% normal goat serum) and placed on ice. High-intensity illumination was provided by a 1,000-W halogen projector lamp giving an intensity of 1,800 μmol photons m^−2^ s^−1^ on the vessel surface.

### *Arabidopsis thaliana* section preparation and photobleaching.

A modified protocol based on the work of Paciorek et al. (43) was used to conduct section preparation of *Arabidopsis thaliana*. Rosette leaves of 3-week-old wild-type *A. thaliana* ecotype Columbia-0 were fixed in 4% paraformaldehyde for 60 min under low vacuum. After infiltration, leaves were washed three times (10 min for each washing) with the MTSB buffer {50 mM PIPES [piperazine-*N*,*N*′-bis(2-ethanesulfonic acid)], 5 mM EGTA, 5 mM MgSO_4_, pH 6.9} and incubated for 15 min with the PBS buffer. Leaves were dehydrated by sequential incubation with 25%, 50%, 75%, and 96% ethanol, each for 60 min. Wax was prepared by mixing polyethylene glycol distearate (Sigma-Aldrich) and 1-hexadecanol (Sigma-Aldrich) at a ratio of 9:1. For embedding, samples were incubated with 33%, 50%, 66%, and 100% wax (in ethanol) at 37°C, each for 60 min. Wax-embedded samples were then sliced by a microtome (Leica) into sections with a thickness of 8 µm. Sections were transferred on the surface of double-distilled water (ddH_2_O) at room temperature. Coverslips were placed in ddH_2_O just beneath the sections and then moved upward, which resulted in adhesion of the sections to the coverslip. The obtained coverslip was air dried overnight. Dewaxing and rehydration were carried out with a series of ethanol solutions (99%, 90%, and 50%). Samples were washed with PBS for 20 min, blocked with 2% bovine serum albumin (BSA), and exposed to high light for 60 min (1,800 μmol photons m^−2^ s^−1^ on the vessel surface).

### Fixation and photobleaching of COS-7 cells.

Green monkey kidney COS-7 cells were plated on glass coverslips at 30% confluence. After 16 to 24 h, cells were subjected to fixation and permeabilization as described previously (28). During the blocking step, coverslips with cells facing up were placed on Parafilm and covered with 3% normal goat serum. Blocking and high-light exposure were carried out simultaneously for the high-light-treated COS-7 cells. For the control samples without high-light treatment, only the blocking step was carried out.

### Immunostaining.

After photobleaching, *Prochlorococcus* cells were stained at room temperature for 30 min with an anti-*Anabaena* FtsZ antibody (catalog no. AS07217; Agrisera), which was dissolved in 200 µl water as recommended by the manufacturer and diluted 1:100 in the blocking buffer. This antibody revealed a band of the expected size in the total protein of *Prochlorococcus* MED4 (see Fig. S3 in the supplemental material) and was also shown to react specifically with the FtsZ protein of a closely related *Prochlorococcus* strain, PCC 9511 (44). Cells were washed with PBS three times and incubated at room temperature in the dark for 40 min with anti-rabbit secondary antibody conjugated with Alexa Fluor 750 (Life Technologies) (1:500 dilution in the blocking buffer). Cells were washed with PBS three times, fixed with 4% paraformaldehyde for 15 min at room temperature, washed with PBS three times, and stored at 4°C in PBS until superresolution imaging.

10.1128/mBio.00657-17.3 FIG S3  Western blot of *Prochlorococcus* MED4 protein with an anti-FtsZ antibody. Download FIG S3, PDF file, 0.04 MB. Copyright © 2017 Liu et al. 2017 Liu et al. This content is distributed under the terms of the Creative Commons Attribution 4.0 International license.

The microtubules of COS-7 cells were immunostained as described previously (28) with a primary antibody to β-tubulin (catalog no. T4026; Sigma-Aldrich).

After section preparation and photobleaching, *A. thaliana* samples were incubated with an anti-PIP2a polyclonal antibody (29) for 12 h at 4°C. After washing with MTSB three times (10 min for each washing), samples were incubated for 5 h at 4°C with an anti-rabbit secondary antibody conjugated with Alexa Fluor 647 (Life Technologies). Samples were washed three times again with MTSB and then were used for STORM imaging.

### Superresolution microscopy with STORM.

A coverslip was assembled in an imaging chamber, with cells immersed in freshly prepared imaging buffer [50 mM Tris (pH 8.0), 10% (wt/vol) glucose, 560 μg/ml glucose oxidase, 57 μg/ml catalase, 2 mM cyclo-octatetraene, 25 mM tris(2-carboxyethyl)phosphine (TCEP), 1 mM ascorbic acid, and 1 mM methyl viologen]. For superresolution microscopy with STORM (dSTORM), images were acquired with an SRiS microscope (NBI, China), which has been previously reported (45). Each superresolution image was reconstructed from a movie containing 10,000 frames recorded at 33 Hz, during which time the dye molecules briefly cycled between dark and bright status for many iterations in the imaging buffer. This “winking” was used to calculate a two-dimensional Gaussian distribution that was assumed to be centered on the location of a single dye molecule. Moderate-excitation laser intensities (4.5 kW/cm^2^ at 750 nm for Alexa Fluor 750) were applied to minimize photobleaching during imaging. Active sample locking was used to stabilize the sample with 1-nm accuracy during imaging. Three-dimensional STORM was achieved by using optical astigmatism (6). Images were processed and analyzed by the Rohdea 1.5 software (NBI, China) and reconstructed with the ImageJ software (National Institutes of Health, USA). 3D rotation films were recorded with the ImageJ plug-in 3D viewer.

To calculate the spatial resolutions of STORM images, we took 20,000 frames of the 100-nm gold particles. Photon count distribution of the gold particles was plotted, and the mean photon count was 5,473. The localization precision was calculated as σ_*xy*_ = 4.07 nm and σ_*z*_ = 17.7 nm. The spatial resolutions can be considered the full width at half maximum (FWHM) of the Gaussian localization precision, which is 2.35 times the localization precision. Using this method, the spatial resolutions of our STORM images were calculated to be 9.6 nm in the *x-y* plane and 41.6 nm in the *z* axis.

### Western blotting.

Log-phase *Prochlorococcus* MED4 cultures were spun down. Cells were resuspended in 50 mM Tris and 500 mM NaCl (pH 8.0) and were lysed by bead beating. Soluble proteins from 7 ml culture were separated by 12% SDS-PAGE and blotted for 1.5 h to polyvinylidene difluoride (PVDF) using tank transfer. Blots were blocked with 5% milk for 1 h at room temperature and then incubated overnight with the FtsZ primary antibody (1:15,000 dilution) at 4°C with agitation. The antibody solution was decanted, and the blot was rinsed briefly twice, then washed once for 15 min and 3 times for 5 min in TBS-T (Tris-buffered saline with Tween 20, pH 8.0) at room temperature. The blot was incubated with the secondary antibody (anti-rabbit IgG conjugated with horseradish peroxidase; Agrisera) at a dilution of 1:25,000 in TBS-T for 1 h at room temperature. The blot was washed as described above and developed for 5 min with enhanced chemiluminescence according to the manufacturer’s instructions.

### Data availability.

STORM images used in this study will be provided upon request.
