# Supplementary material for: Three-Dimensional Superresolution Imaging of the FtsZ Ring during Cell Division of the Cyanobacterium Prochlorococcus
Source: mBio. 2017 Nov 21;8(6):e00657-17. doi: 10.1128/mBio.00657-17 (PMC5698547; doi:10.1128/mBio.00657-17)
Supplement: FIG S1 [file mbo006173604sf1.pdf]

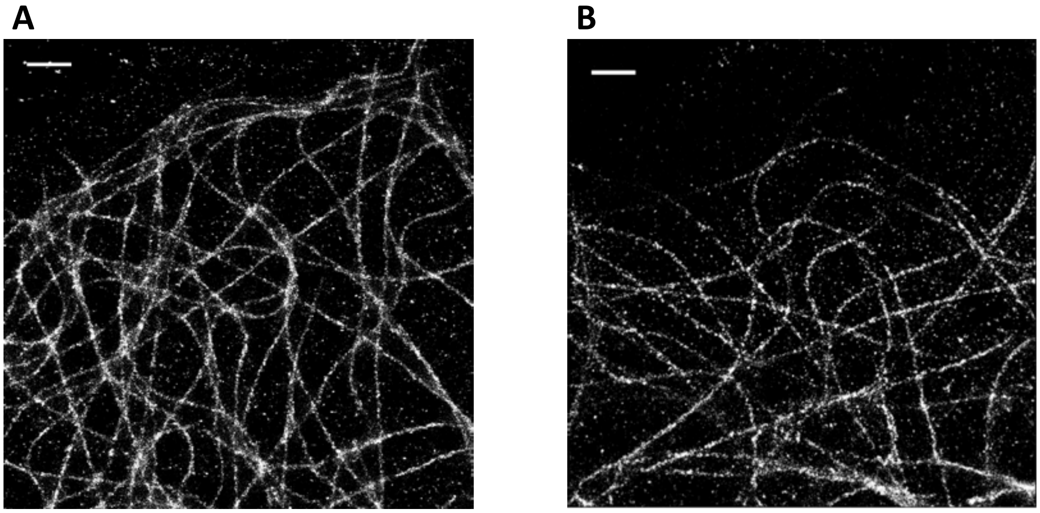

Fig S1. STORM imaging of microtubules in COS-7 cells

COS-7 cells were exposed to while light of high intensity for 60 min (**A**) or without high light exposure (**B**). Following two published studies (Bates et al., 2007; Huang et al., 2008), microtubules of COS-7 cells were immunostained with a primary antibody against β-tubulin. Scale bars, 1 µm.
