# Supplementary material for: Three-Dimensional Superresolution Imaging of the FtsZ Ring during Cell Division of the Cyanobacterium Prochlorococcus
Source: mBio. 2017 Nov 21;8(6):e00657-17. doi: 10.1128/mBio.00657-17 (PMC5698547; doi:10.1128/mBio.00657-17)
Supplement: FIG S2 [file mbo006173604sf2.pdf]

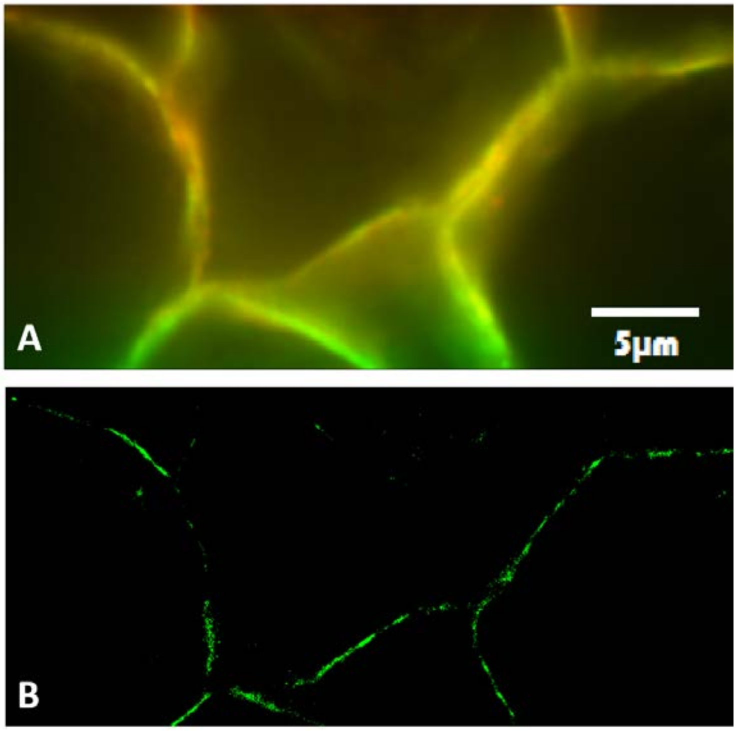

Fig S2. STORM imaging of plant plasma membrane intrinsic protein 2a (PIP2a, AT3G53420)

After section preparation and photobleaching, *A. thaliana* samples were immunostained with an anti-PIP2a antibody (Qing et al., 2016). (**A**) Wide field image. (**B**) STORM image.
