# Supplementary material for: Three-Dimensional Superresolution Imaging of the FtsZ Ring during Cell Division of the Cyanobacterium Prochlorococcus
Source: mBio. 2017 Nov 21;8(6):e00657-17. doi: 10.1128/mBio.00657-17 (PMC5698547; doi:10.1128/mBio.00657-17)
Supplement: FIG S3 [file mbo006173604sf3.pdf]

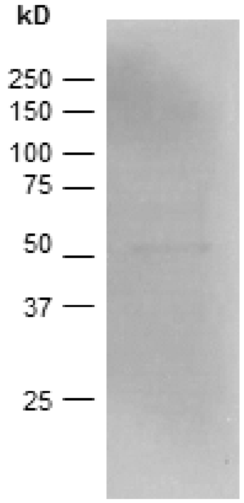

Fig S3. Western blot of *Prochlorococcus* MED4 protein with an anti-FtsZ antibody

Total protein of *Prochlorococcus* MED4 was extracted and separated. The FtsZ protein was detected with an anti-*Anabaena* FtsZ antibody. A band of the expected size (~50 kD) was detected, which is similar to the FtsZ band size of *Synechococcus elongatus* PCC 7942 (Miyagishima et al., 2005). The Precision Plus Protein Standards (Bio-Rad) were used in the blot.
